# Supplementary material for: Extracts and Scirpusin B from Recycled Seeds and Rinds of Passion Fruits (Passiflora edulis var. Tainung No. 1) Exhibit Improved Functions in Scopolamine-Induced Impaired-Memory ICR Mice
Source: Antioxidants (Basel). 2023 Nov 29;12(12):2058. doi: 10.3390/antiox12122058 (PMC10741041; doi:10.3390/antiox12122058)
Supplement: Supplementary file 1 [file antioxidants-12-02058-s001.zip › antioxidants-2730299-supplementary.pdf]

Extracts and Scirpusin B from Recycled Seeds and Rinds of Passion Fruits (*Passiflora edulis* var. Tainung No. 1) Exhibit Improved Functions in Scopolamine-Induced Impaired Memory ICR Mice

Yi-Yan Sie[1,†], Liang-Chieh Chen[2], Cai-Wei Li[3,†], Ching-Chiung Wang[1,3,4,5], Cai-Jhen Li[3], Der-Zen Liu[6], Mei-Hsien Lee[1,3], Lih-Geeng Chen[4,7,*], and Wen-Chi Hou[1,3,*]

[1] Ph.D. Program in Clinical Drug Development of Herbal Medicine, College of Pharmacy, Taipei Medical University, Taipei 110, Taiwan; d339110003@tmu.edu.tw (Y.-Y. S.); crystal@tmu.edu.tw (C.-C. W.); lmh@tmu.edu.tw (M.-H.L); wchou@tmu.edu.tw (W.-C. H.)

[2] School of Medicine, College of Medicine, National Sun Yat-sen University, Kaohsiung 804, Taiwan; lcchen0908@mail.nsysu.edu.tw

[3] Graduate Institute of Pharmacognosy, Taipei Medical University, Taipei 110, Taiwan; licaiwei@tmu.edu.tw (C.-W. L.); crystal@tmu.edu.tw (C.-C. W.); m303108003@tmu.edu.tw (C.-J. L.); lmh@tmu.edu.tw (M.-H.L); wchou@tmu.edu.tw (W.-C. H.)

[4] Traditional Herbal Medicine Research Center, Taipei Medical University Hospital, Taipei, Taiwan; crystal@tmu.edu.tw (C.-C. W.); lgchen@mail.ncyu.edu.tw (L.-G. C.)

[5] School of Pharmacy, Taipei Medical University, Taipei 110, Taiwan; crystal@tmu.edu.tw

[6] Graduate Institute of Biomedical Materials and Tissue Engineering, Taipei Medical University, Taipei 110, Taiwan; tonyliu@tmu.edu.tw

[7] Department of Microbiology, Immunology and Biopharmaceuticals, College of Life Sciences, National Chiayi University, Chiayi 600, Taiwan; lgchen@mail.ncyu.edu.tw

* Correspondence: lgchen@mail.ncyu.edu.tw (L.-G. C); wchou@tmu.edu.tw (W.-C.H.)

† These two authors contributed equally to this work.

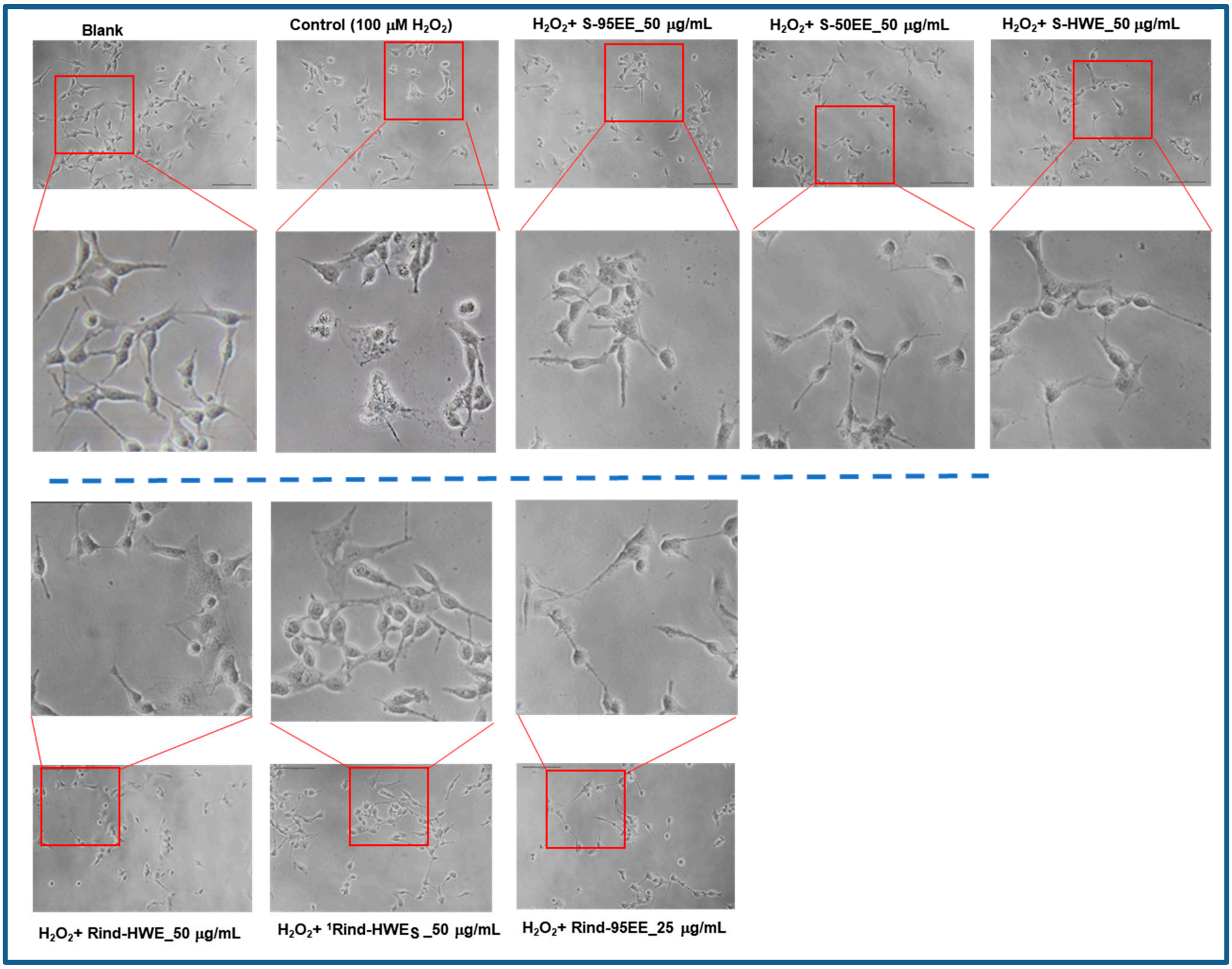

Figure S1. Effects of seed extracts (50 μg/mL) and rind extracts (25 or 50 μg/mL) of passion fruits (*Passiflora edulis* var. Tainung No. 1) on cell morphologies of 100 μM hydrogen peroxide-treated SH-SY5Y cells. The cells (1× 10^4 cells/well) were seeded onto a 96-well microplate and cultured in a DMEM/F-12 medium containing 10% FBS for 24 h at 37 °C under a humidified atmosphere and 5% CO_2. The treated medium were removed, washed with PBS, and 100 μM hydrogen peroxide in PBS were added to the medium for another 24 h culture. The cell morphologies were photographed (400-fold magnifications) and local zooms of images (red square frame) using an inverted microscope (ECLIPSE TS100, Nikon Instruments Inc., Tokyo, Japan). S, de-oiled seed powders; 95EE, 95% ethanol extracts; 50EE, 50% ethanol extracts; HWE, hot-water extracts.

[1]Ethanol was added to Rind-HWE solution to final 80%, the precipitates were removed, and the supernatants were concentrated under reduced pressures and then lyophilized as the Rind-HWE$_S$.
